# Human Monoclonal Antibodies against Glucagon Receptor Improve Glucose Homeostasis by Suppression of Hepatic Glucose Output in Diet-Induced Obese Mice

**DOI:** 10.1371/journal.pone.0050954

**Published:** 2012-12-03

**Authors:** Wook-Dong Kim, Yong-ho Lee, Min-Hee Kim, Sun-Young Jung, Woo-Chan Son, Seon-Joo Yoon, Byung-Wan Lee

**Affiliations:** 1 Department of New Drug Discovery, Neopharm Co., Ltd., Daejeon, Korea; 2 Department of Internal Medicine, Yonsei University College of Medicine, Seoul, Korea; 3 Department of Medicine, Graduate School Yonsei University, Seoul, Korea; 4 Department of Pathology, Asan Medical Center, University of Ulsan, College of Medicine, Seoul, Korea; University of Ulster, United Kingdom

## Abstract

**Aim:**

Glucagon is an essential regulator of hepatic glucose production (HGP), which provides an alternative therapeutic target for managing type 2 diabetes with glucagon antagonists. We studied the effect of a novel human monoclonal antibody against glucagon receptor (GCGR), NPB112, on glucose homeostasis in diet-induced obese (DIO) mice.

**Methods:**

The glucose-lowering efficacy and safety of NPB112 were investigated in DIO mice with human GCGR for 11 weeks, and a hyperinsulinemic-euglycemic clamp study was conducted to measure HGP.

**Results:**

Single intraperitoneal injection of NPB112 with 5 mg/kg effectively decreased blood glucose levels in DIO mice for 5 days. A significant reduction in blood glucose was observed in DIO mice treated with NPB112 at a dose ≥5 mg/kg for 6 weeks, and its glucose-lowering effect was dose-dependent. Long-term administration of NPB112 also caused a mild 29% elevation in glucagon level, which was returned to the normal range after discontinuation of treatment. The clamp study showed that DIO mice injected with NPB112 at 5 mg/kg were more insulin sensitive than control mice, indicating amelioration of insulin resistance by treatment with NPB112. DIO mice treated with NPB112 showed a significant improvement in the ability of insulin to suppress HGP, showing a 33% suppression (from 8.3 mg/kg/min to 5.6 mg/kg/min) compared to the 2% suppression (from 9.8 mg/kg/min to 9.6 mg/kg/min) in control mice. In addition, no hypoglycemia or adverse effect was observed during the treatment.

**Conclusions:**

A novel human monoclonal GCGR antibody, NPB112, effectively lowered the glucose level in diabetic animal models with mild and reversible hyperglucagonemia. Suppression of excess HGP with NPB112 may be a promising therapeutic modality for the treatment of type 2 diabetes.

## Introduction

Though type 2 diabetes (T2D) is a multifactorial syndrome of metabolic dysregulation, current pharmacologic strategies for T2D are focused on the progressive decline in pancreatic β-cell functions with diminished tissue responses to insulin (insulin resistance), closely linked with abnormalities in carbohydrate, fat, and protein metabolism [1]. In normal glucose and type 2 diabetic conditions, insulin secretory function of β-cells in response to surged carbohydrate from meal loading, and peripheral insulin sensitivity are main gluco-insulin axis [2]. It is well known that impaired α-cell function can lead to excessive glucagon release during postabsorptive and fasting states, which contributes to the development and progression of hyperglycemia [3]. In this regard, a therapeutic approach to overcome the uncontrolled excessive postabsorptive and fasting hepatic glucose production (HGP) as well as insulin resistance in the context of progressive islet dysfunction leading to hyperglycemia [4] might be more physiologic to achieve glycemic control.

In type 2 diabetics, the metabolic homeostasis in glucose and glucagon are largely characterized by high *α*-cell secretion of plasma glucagon, especially in subjects with poorly controlled and insulinopenic status [5] or impaired counter-regulatory response of glucagon to hypoglycemia [6]. These inappropriate balances between postabsorptive and fasting glucagon secretion drive excessive HGP in T2D. However, therapeutic modalities targeting the underlying defects in oversecretion of glucagon that cause these abnormal physiologic responses have not been well investigated. To date, several glucagon antagonists have been reported to regulate glucagon-mediated signal pathway in diabetic animals. Treatment with non-peptide compounds that antagonize GCGR inhibited hepatic glucose production and decreased blood glucose levels [7], [8]. Antisense oligonucleotides significantly improved glucose tolerance and hyperglycemia in various diabetic models such as *db*/*db*, *ob*/*ob* mice and ZDF rats [9], [10]. Recently, monoclonal antibodies targeting GCGR have been also developed to achieve improvement in glycemic control [11]–[13]. Although many reports with glucagon antagonists showing glucose-lowering efficacy in various animal models have been published, there is no clinically available glucagon antagonists for humans with diabetes so far, indicating that continuous efforts are highly required to develop novel drugs targeting glucagon signaling pathways.

The current report describes a series of studies designed to examine the effects of treatment with a novel, human monoclonal antibody against glucagon receptor (GCGR) on glucose reduction as well as the metabolic consequences and mechanism of potential compensatory responses stemming from GCGR antibody treatment.

## Materials and Methods

### Establishment of Recombinant Cell Lines Expressing GCGR

Stable cell lines expressing GCGR were established according to similar procedures described previously [11]. Briefly, recombinant GCGR cDNAs originating from murine, cynomolgus monkey, and human were subcloned into expression vector plasmids containing a selectable antibiotic gene. After transfected cells were subcloned under appropriate antibiotic selection, glucagon-induced cyclic AMP accumulation and specific ^125^I-glucagon binding were measured for screening.

The cell line expressing a high level of hGCGR was developed by transfecting a CMV promoter-driven expression vector with full-length hGCGR cDNA into AM1D cells. GCGR mRNA level and specific ^125^I-glucagon binding were then measured to select a stable subclone. After an expression construct was created by combining hGCGR with human recombinant GFP (Stratagene, La Jolla, CA, USA) at the C-terminus, a stable cell line with hGCGR-GFP was generated in 293T cells (293-HEK-hGCGR-GFP cells). FACS was performed to select and enrich transfected cells with high expression of GFP.

### Development and Selection of Anti-GCGR Antibodies

To produce high-affinity human monoclonal antibodies to the hGCGR from XenoMouse®(Amgen British Columbia, Inc, Burnaby, BC), we chose to use the N-terminal extracellular domain of the GCGR fused to an Fc fragment, whole-cell membranous fractions from GCGR-expressing cell lines, and peptides corresponding to the extracellular regions. Supernatants of hybridomas were tested for specific binding to hGCGR using fluorometric microvolume assay technology. In total, 122 monoclonal antibodies that proved to be bound specifically to hGCGR were initially selected. Crude hybridoma supernatants of the specific antibodies were screened for their ability to suppress glucagon-induced cAMP production in hGCGR recombinant cells. Antibodies with antagonistic activities were isolated from supernatants, and inhibitory properties were investigated at a 2 µM final concentration. We used phage display methods using immuno-tube to generate and select specific anti-GCGR antibodies. The surface of each immuno-tube was coated with either positive or negative antigen against hGCGR (1 mL at concentration of 10 µg/mL) at 37°C for 1 hr. Then libraries were blocked with solution of 2% skim milk in PBS (MPBS) to adjust the titer of those into the concentration of 10^11 ^CFU/µL. Antigen-coated immuno-tubes were also blocked with MPBS for 1 hr at room temperature. Each library blocking solution was transferred into negative antigen-coated immuno-tubes and incubated for 1 hr at room temperature after MPBS was removed from those tubes. Then, library blocking solution from negative antigen-coated tubes was transferred into MPBS removed immuno-tubes coated with positive antigen and incubated for 1 hr at room temperature. After removal of library blocking solution, positive antigen-coated immuno-tubes were washed with MPBS for twice and PBST (0.05% tween 20) for 7 times, followed by repeated washing with PBS for 3 times to eliminate unbound phage libraries from the tube. Washed tubes were eluted with 1 mL 100 mM TEA (pH 12) for 10 min at room temperature and neutralized with 150 uL 1 M Tris-HCl (pH 7.4). Eluted phage was infected into 10 mL XL1 Blue (*Escherichia coli*) at 37°C for 1 hr and the culture was centrifuged at 5000g for 15 min at room temperature. The pellet was resuspended in 900 mL 2TY phage medium and spread in agarose plates for overnight incubation at 37°C. A colony from a streaked 2TY agarose plate of XL1 Blue was inoculated into 50 mL 2TY medium and grown overnight at 37°C. When an optical density 600 nm (OD600) of 0.8 is reached, helper phage (M13-K07) was infected into the culture and incubated at 37°C for 1 hr with shaking (at 100 rpm). After centrifuge at 5000 g, pellets were resuspended into 50 mL 2TY medium and grown overnight at 33°C. Supernatant was isolated after centrifuge at 12,000 g for 15 min and second round panning was repeatedly conducted with using this supernatant. When performing second round panning, selected single colony was inoculated into 96 well plates and high affinity antibodies with positive antigen were determined by using ELISA methods [14]. NPB112 was subsequently cloned for recombinant expression as human IgG_2_.

### Antibody Activity Assay: cAMP Assay

After resuspension of 293-HEK-hGCGR-GFP cells at 5×10 cells/ml in assay buffer (PBS+0.1% bovine serum albumin) with 1 mM 3-isobutyl-1-methylxanthine (IBMX, Sigma-Aldrich, St. Louis, MO), the cell suspension was mixed with cAMP-d2 working solution containing lyophilized powder dissolved in distilled water. Serially diluted test antibodies were added to recombinant cells and incubated at 37°C for 20 min. Cells were treated with fresh glucagon solution (Bachem, Bubendorf, Switzerland) at a final concentration of 50 pM in the presence of IBMX and incubated at 37°C for 20 min. The cell stimulation was stopped by adding an equal amount of lysis buffer containing cAMP-XL665 and anti-cAMP-cryptate. After shaking the plate for 1 h at room temperature, the cAMP levels were quantified using a Cisbio cAMP dynamic 2 kit (Cis Bio International, Gif-sur-Yvette, France). The homogeneous time-resolved fluorescence (HTRF) 2-step protocol was performed according to the manufacturer’s instructions, and the RUBYstar (BMG Labtech, Durham, NC) instrument was used to detect the HTRF signal. Nonlinear regression analyses of resulting concentration curves were plotted using GraphPad Prism (GraphPad Software, Inc., San Diego, CA).

#### Antibody affinity assay: cell ELISA

Ninety-six wells were coated with test antibodies (anti-human Lambda antibodies) by adding the solution at a concentration of 2 µg/ml in PBS to each well, and the plates were incubated overnight at 4°C. Standard human IgG was used as the internal control. Plates were then blocked with 100 µl of 2% milk in PBS for 1 h at room temperature. Then, 293-HEK-hGCGR-GFP cells were added into each well, incubated for 1 h at 37°C and then washed 3 times with PBS-0.01% Tween. Anti-human IgG-horseradish peroxidase (HRP) conjugate (1∶10000, Sigma-Aldrich, SL) was added to the plate and incubated for 1 h at room temperature. After washing 3 times with PBS-0.01% Tween, plates were filled with 50 µl of TMB substrate (Kirkegaard and Perry Laboratories, KPL, Gaithersburg, MD) solution according to the manufacturer’s instructions and measured on the luminometer (SPECTRA max 340PC, Molecular Devices Corporation, Sunnyvale, CA).

#### Pharmacokinetic analysis

The pharmacokinetics of NPB112 were conducted in male Sprague-Dawley (SD) rats of which right jugular veins were cannulated with catheters. A total of 10 rats were divided into two groups (n = 5, each) and NPB112 at 5 mg/kg was intravenously or intraperitoneally administered into each group, respectively. Blood samples for pharmacokinetic analysis were collected from each animal in the 5 mg/kg dose groups on 6 hours and days 1, 2, 3, 4, 5, 6, 7, and 10. The concentrations of NPB112 in plasma samples were measured by a sandwich enzyme-linked immunosorbent assay using rabbit anti-human-lambda antibodies and horseradish peroxidase-conjugated mouse anti-human IgG antibodies. Pharmacokinetic parameters including T_1/2_ and area under the concentration-time curve (AUC) were estimated from NPB112 plasma concentration data via noncompartmental analysis using Kinetica™ software version 4.4.1 (Pharsight, Mountain View, CA) and the linear/logarithmic trapezoidal method.

#### Animals and diets

All mouse experiments were conducted at NeoPharm Co., LTD. (Daejon, Korea), and all procedures were approved by the Institutional Animal Care and Use Committee named “Neopharm an animal experiment committee” (The animal experiment identification number 33th). All mice were anesthetized by intraperitoneal injection with 0.1cc/100g zoletil. Twelve–week–old male C57BL/6N mice with human GCGR were obtained from the Institute of Medical Science, University of Tokyo (Japan). The mice were maintained at ambient temperature (22°C ±1°C) with 12-hour light-dark cycles and free access to water and food. Human GCGR mice were fed a 60% kcal% fat diet (HFD, D12492, Research Diets, NJ) for 8 weeks to induce obesity. The composition of the HFD was approximately 5.24 kcal/g, and the relative amounts were 26.2 gm% protein, 26.3 gm% carbohydrate and fat 34.9 gm% fat.

### Glucose Tolerance Tests

An intraperitoneal glucose tolerance test (IPGTT) was performed after an overnight fast for 14 hours in the hGCGR mice at 20 weeks of age. NPB112 at a dose of 5.0 mg/Kg was injected in each mouse 48 hours before the IPGTT. After 1 g/kg of glucose was intraperitoneally injected, blood glucose levels were measured at 30, 60, 90 and 120 min using a glucose analyzer (Accu-Check; Roche Diagnostics, Basel, Switzerland).

### Efficacy Studies in hGCGR Mice with HFD

1) Effect of short-term treatment with NPB112 on glucose reduction and body weight changes.

Before starting the NPB112 injection, blood glucose levels and body weight were measured in 20-week-old male C57BL/6N mice fed a HFD for 8 weeks (n = 9) in order to evaluate the short-term glucose reduction and body weight changes. On day 1 (time 0), all mice were bled for blood glucose measurement and were injected intraperitoneally with vehicle or NPB112 at 1.2 and 5 mg/kg at the dose volume of 10 ml/kg. Subsequent blood glucose measurements (Accu-Check Inform, Roche Diagnostics) were performed via tail vein collection at 1, 2, 3, 5, and 7 days after injection.

2) Effect of long-term treatment with NPB112 on glucose reduction and body weight changes.

To evaluate the 6-week, long-term glucose reduction and body weight changes, 20-week-old male C57BL/6N mice fed a HFD for 8 weeks were classified into 5 groups. Each group consisted of 24 mice and was administrated vehicle or NPB112 at 0.3 mg/kg, 1.0 mg/kg, 3.0 mg/kg and 10.0 mg/kg, respectively, twice a week for 6 consecutive weeks (total of 12 injections). After the 6-week treatment with NPB112, mice were observed for a4 week drug washout periods. Subsequent measurements of blood glucose and body weight were performed weekly for 11 weeks. Serum levels of insulin and glucagon were repeatedly measured at 0, 2, 4, 7, 9 and 11 weeks after treatment.

### Hypoglycemia in ICR Mice

To determine whether NPB112 cells induce hypoglycemia in normal conditions, male ICR mice (Jackson Laboratory, Bar Harbor, ME) were prepared. ICR mice were randomly assigned at 13 weeks of age into 3 groups; 1) control group treated with vehicle (150 µL of PBS buffer, n = 7), 2) low dose group with NPB112 (5.0 mg/kg, n = 7), 3) high dose group with NPB112 (10.0 mg/kg, n = 7). Forty-eight hours after intraperitoneal administration with the drug or vehicle, ICR mice were fasted for 16 hours. Then, blood glucose levels were measured using a glucose analyzer (Accu-Check Inform, Roche Diagnostics).

### Biochemical Analyses

Blood samples were obtained by cardiac puncture and immediately centrifuged at 5000g for 5 minutes. After 0, 2, and 4 weeks from discontinuation of NPB112, levels of aspartate aminotransferase (AST), alanine aminotransferase (ALT), total cholesterol, triglycerides, non-esterified fatty acids (NEFA), and hemoglobin were determined using a HITACHI 7180 automatic analyzer (Daiichi, Japan). Serum levels of glucagon and insulin were measured using an ELISA kit (ALPCO Diagnostics, Windham, NH).

### Hyperinsulinemic-euglycemic Clamp Study to Measure Hepatic Glucose Output

Twelve-week-old male C57BL/6N mice were fed a high-fat diet for 8 weeks (n = 5 per group). Indwelling catheters were positioned into the right internal jugular vein extending to the right atrium and mice were stabilized for 7 days to initiate the hyperinsulinemic-euglycemic clamp studies. After mice were fasted during overnight, [3-^3^H]glucose (HPLC purified; Perkin Elmer Inc., Norwalk, CT) was infused at a rate of 0.05 µCi/min for 2 hours to examine the basal glucose turnover. Following the basal period, hyperinsulinemic-euglycemic clamps were operated for 120 minutes with a primed/continuous infusion of human insulin (100∼150 pmol/kg prime, 15∼18 pmol/kg/min infusion; Novo Nordisk, Bagsvaerd, Denmark) in order to raise plasma insulin levels to within the physiological range. Blood samples (10 µl) were collected at 10- to 20-minute intervals for the immediate measurement of plasma glucose, and 20% dextrose was infused at variable rates to maintain plasma glucose at basal levels (∼6.7 mM). To assess insulin-stimulated whole-body glucose fluxes, [3-^3^H]glucose was infused at a rate of 0.1 µCi/min throughout the clamps, and 2-deoxy-D-[1-^14^C] glucose (2-[^14^C]DG, HPLC purified; PerkinElmer) was injected as a bolus 75 minutes into the clamping to estimate the rate of insulin-stimulated tissue glucose uptake. Blood samples were obtained at the end of the basal period and during the last 45 minutes of the clamping to measure plasma [^3^H]glucose and [^14^C]glucose activities. Blood samples were collected repeatedly to measure plasma insulin concentrations at the end of the basal and clamp periods. At the end of the clamp experiments, pentobarbital sodium was injected into mice, and tissues were collected for biochemical measurements within 4 minutes. Mouse tissues were dissected and frozen immediately using liquid N2–cooled aluminum blocks, and stored at –80°C for subsequent analysis.

### Statistical Analyses

Data are presented as mean ± SEM. Data were compared using the Student’s t test or analysis of variance using the SPSS 16.0 program (SPSS Institute, Chicago, IL). Statistical significance was defined as a P-value less than 0.05.

## Results

### NPB112 is a Fully Humanized Antibody of hGCGR with Good Affinity and Potent Antagonistic Activity in vitro

**Figure 1:**
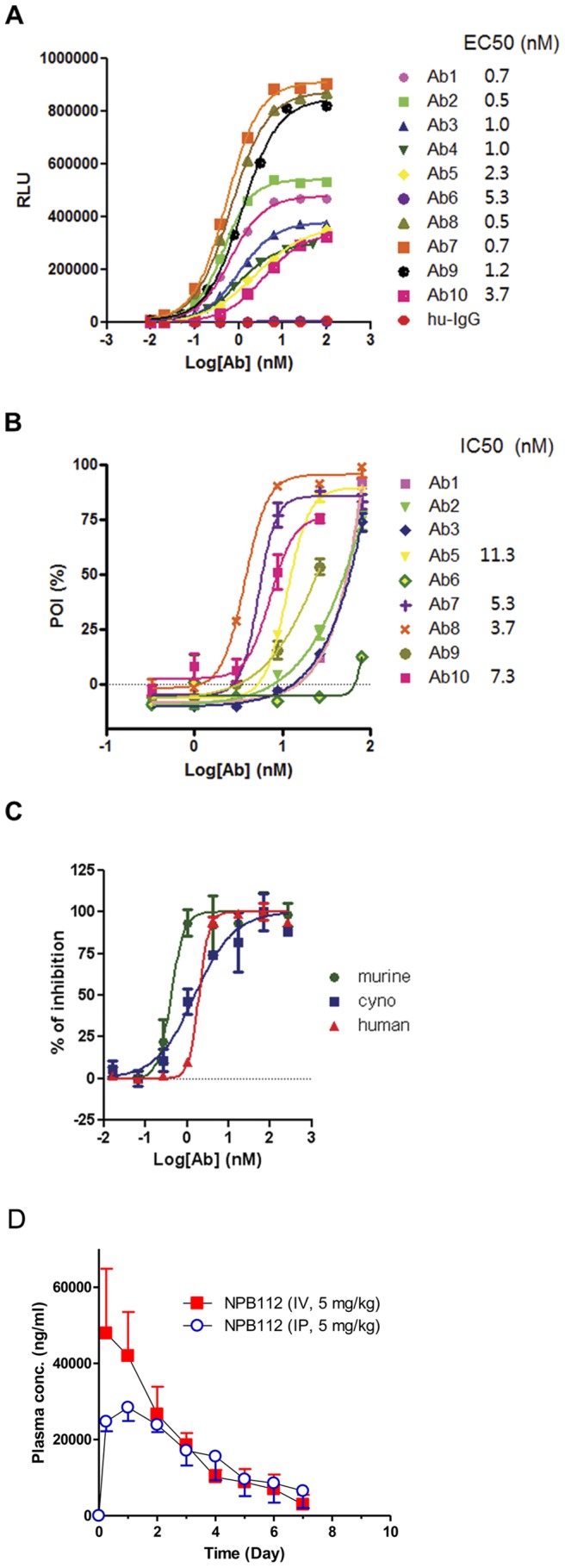
*In vitro* study of the affinity and functional activity of antibodies. A. ELISA of the hGCGR-expressing cell line with candidate antibodies including NPB112 (designated as Ab8). B. Cell-based cAMP assay using hGCGR-expressing cell lines with candidate antibodies including NPB112 (designated as Ab8). C. Cell-based cAMP assay with NPB112 using recombinant cell lines expressing different GCGRs from human, murine, and cynomolgus monkey. D. Plasma concentration-time profiles of NPB112 in male Sprague-Dawley rats following a single intravenous or intraperitoneal administration of 5 mg/kg NPB112. Data are expressed as mean ± S.D. (n = 5/time point/group).

Among 122 human monoclonal immunogammaglobulin 2 varieties that have been confirmed to specifically bind to native hGCGR, NPB112 was identified and selected for further experiments. To evaluate the affinity to hGCGR and the antagonistic activity of the antibody, cell-based ELISA and cAMP assay were performed using a recombinant cell line expressing hGCGR (293-HEK-hGCGR-GFP cells). Figure 1A shows that NPB112, designated as Ab8, had the highest binding affinity to hGCGR with an EC_50_ of 0.5 nM. NPB112 effectively inhibited the production of cAMP by glucagon with an IC50 of 3.7 nM against hGCGR in a cell-based cAMP assay (Figure 1B). Furthermore, NPB112 demonstrated fairly good antagonistic activity against glucagon receptors from human, murine, and cynomolgus monkey (Figure 1C), with IC50s of 1.92, 1.53 and 0.45 nM, respectively. According to the sequence analysis of NPB112, neither a predicted glycosylation site nor extra cysteine was found in the variable regions of the antibody (data not shown). Pharmacokinetic analyses conducted in SD rats showed that both intravenous and intraperitoneal administration displayed similar pharmacokinetic properties (Table 1). The mean half-life of NPB112 was 86.8 h in intraperitoneally injected rats and 49.8 h in intravenously injected rats with low systemic clearance (0.001 l/hr/kg). After intraperitoneal administration of NPB112 in rats, C_max_ value was achieved on day 2 (T_max_ = 24 h) and peritoneal bioavailability was fairly good (F = 103.3%).

**Table 1:** Pharmacokinetic parameters of NPB112 after single intravenous or intraperitoneal administration of 5 mg/kg NPB112 to Sprague-Dawley rats.

	Intravenous administration(n = 5)	Intraperitoneal administration(n = 5)
T_max_ (h)	NA	24.0±0.0
C_max_ (ug/ml)	NA	28.4±3.5
AUC_last_ (ug•h/ml)	3354.2±802.9	2766.1±593.5
Clearance (l/h/kg)	0.001±0.0004	NA
T_1/2_ (h)	49.8±28.2	86.8±52.8
F (%)	NA	103.3±45.5

NA: not applicable.

### Effect of Short-term Treatment with NPB112 on Glucose Reduction in hGCGR Mice with HFD (10 days)

**Figure 2:**
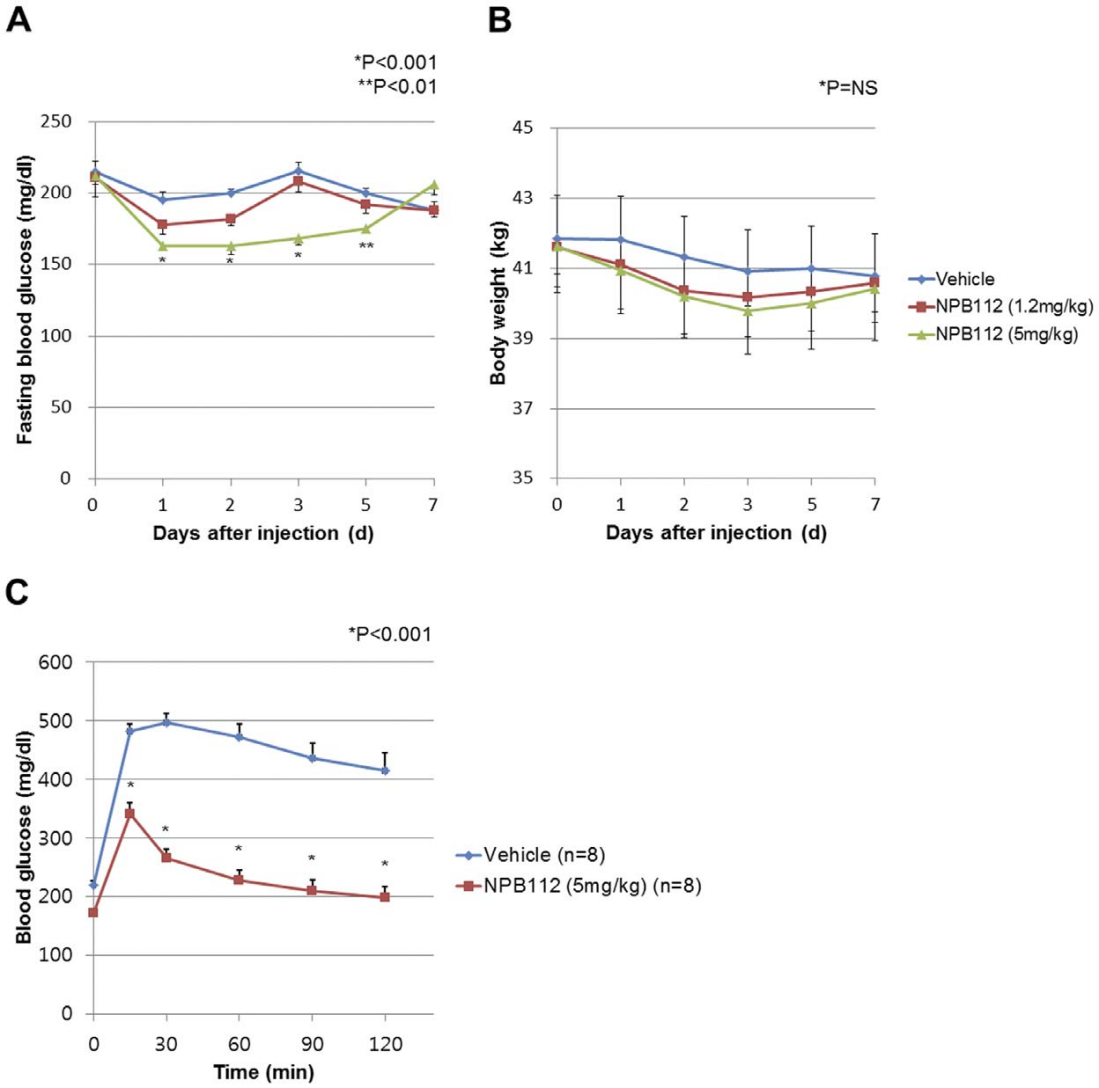
Effect of NPB112 on blood glucose, body weight and GTT in DIO mice. A. Fasting blood glucose levels before (0 day) and after single injection of vehicle or NPB112 in male DIO mice (n = 9∼10 per group). Values are expressed as mean ± SEM *, p<0.001 compared to control mice with vehicle. **, p<0.01 compared to control mice with vehicle. B. Body weight before (0 day) and after single injection of vehicle or NPB112 in male DIO mice (n = 9∼10 per group). C. IPGTT performed after a 14-h fast, 2 days after single injection of vehicle or NPB112 in male DIO mice (n = 8 per group). Blood glucose levels at baseline, 30, 60, 90 and 120 min after glucose administration were measured. Values are expressed as mean ± SEM *, p<0.001 compared to control mice with vehicle.

Twenty-week-old diet-induced obese (DIO) mice fed an HFD for 8 weeks received NPB112 at 1.2 or 5 mg/kg to evaluate whether NPB112 lowers blood glucose level *in vivo*. After single injection of NPB112, glucose levels and body weights were measured for 7 days in DIO mice. Compared to control mice, dose-dependent reduction in blood glucose level was observed; DIO mice treated with the highest dose of NPB112 (5 mg/kg) had a significant decrease in plasma glucose on the first day after injection (Figure 2A). Blood glucose level returned to baseline (glucose level at day 0) at day 3 with a 1.2 mg/kg dose, while the glucose-lowering effect was maintained for 5 days, returning to baseline at 7 days, with a 5 mg/kg dose of NPB112. Body weight was slightly reduced after injection of NPB112; however, there was no significant difference in body weight changes during the study period (Figure 2B).

To investigate the effect of NPB112 on glucose tolerance in DIO mice, we performed IPGTT in DIO mice on day 2 after a single subcutaneous injection of NPB112 at a dose of 5 mg/kg. Figure 2C shows that a 5 mg/kg dose of NPB112 significantly improved glucose tolerance in DIO mice (P<0.001 versus vehicle-treated mice at 30, 60, 90, 120 min).

### Effect of Long-term Treatment with NPB112 on Glucose Lowering in hGCGR Mice with HFD (11 weeks)

**Figure 3:**
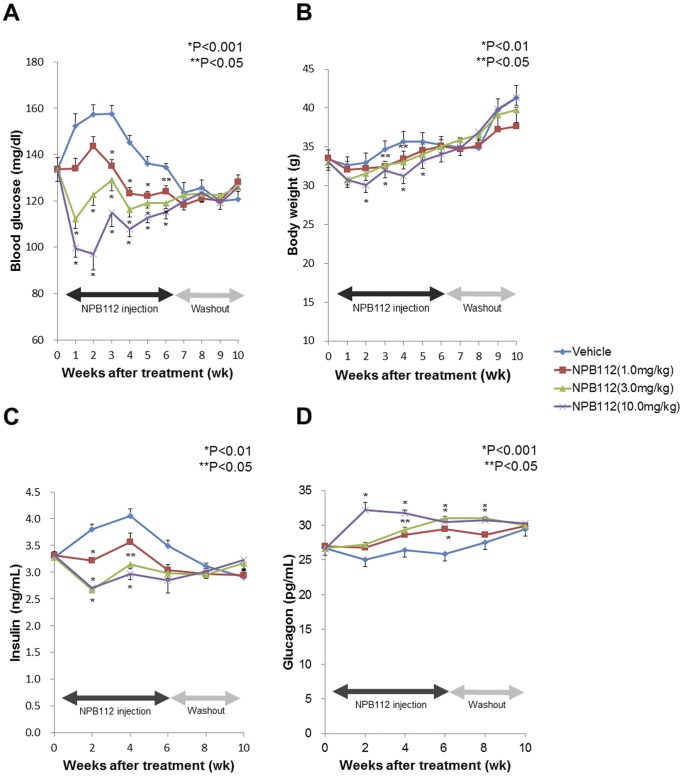
Effect of NPB112 on levels of blood glucose (A), body weight (B), insulin (C) and glucagon (D) in DIO mice before and every week after injection of vehicle of NPB112 for 6 weeks and 4 weeks of recovery period (n = 24 per group). Values are expressed as mean SEM (A and D) *, p<0.001 and **, P<0.05 compared to control mice with vehicle. (B and C) *, p<0.01 and **, P<0.05 compared to control mice with vehicle.

We then investigated the chronic glucose-lowering effect of NPB112 in DIO mice. After being fed a HFD for 8 weeks, DIO mice received NPB112 at 1.0 mg/kg, 3.0 mg/kg and 10.0 mg/kg twice a week for 6 consecutive weeks (total of 12 injections). The recovery period with no injection of NPB112 lasted another 4 weeks. The blood glucose level and body weight of DIO mice were measured once a week for all 11 weeks of the experiment. In terms of glycemic control, a significant reduction in blood glucose level was observed in DIO mice treated with a high dose of NPB112 (3.0 and 10.0 mg/kg) during the initial 6 weeks, while blood glucose level was gradually increased after 6 weeks of treatment (Figure 3A). The glucose-lowering efficacy of NPB112 in DIO mice was dose-dependent, and no hypoglycemia was observed in any animals at any dose of NPB112 throughout the entire experiment period. With respect to changes in body weight, there was no significant difference in body weight between control and experimental groups during the entire treatment period (Figure 3B). Although DIO mice lost their weight in the first 2 to 3 weeks of experiment, their weight was restored and increased after 3 weeks including the recovery period.

During the NPB112 injection period from 1 to 6 weeks, insulin levels were significantly decreased in DIO mice treated with NPB112 at a 10.0 mg/kg dose compared to control mice (Figure 3C). As shown in Figure 3D, a 10.0 mg/kg dose of NPB112 produced a significant 29% elevation in glucagon level compared to DIO mice treated with vehicle. Despite the decreased insulin levels and increased glucagon levels in the DIO mice treated with NPB112 at 10.0 mg/kg, blood glucose levels did not increase during the experiment. The effect of NPB112 on secretion of insulin and glucagon in DIO mice was dose-dependent, while those effects were both abolished after discontinuation of NPB112 administration.

### Suppression of Hepatic Glucose Output by NPB112 in DIO Mice

To independently assess whole-body and hepatic insulin sensitivities *in vivo*, we performed a hyperinsulinemic-euglycemic clamp study including radioisotope-labeled glucose infusion in DIO mice treated with vehicle or NPB112 at 5.0 mg/kg. Nine-week-old male hGCCR mice were fed HFD for 8 weeks before the clamp study, and a single injection of NPB112 (5.0 mg/kg) was administered to each animal. After ^3^H-glucose infusion into 12-h fasted DIO mice, hepatic glucose output and glucose clearance were measured in DIO mice treated with vehicle or NPB112 at a dose of 5.0 mg/kg. Consistent with our previous results, fasting glucose in NPB112-treated mice decreased by 18% compared with that in control mice.

**Figure 4:**
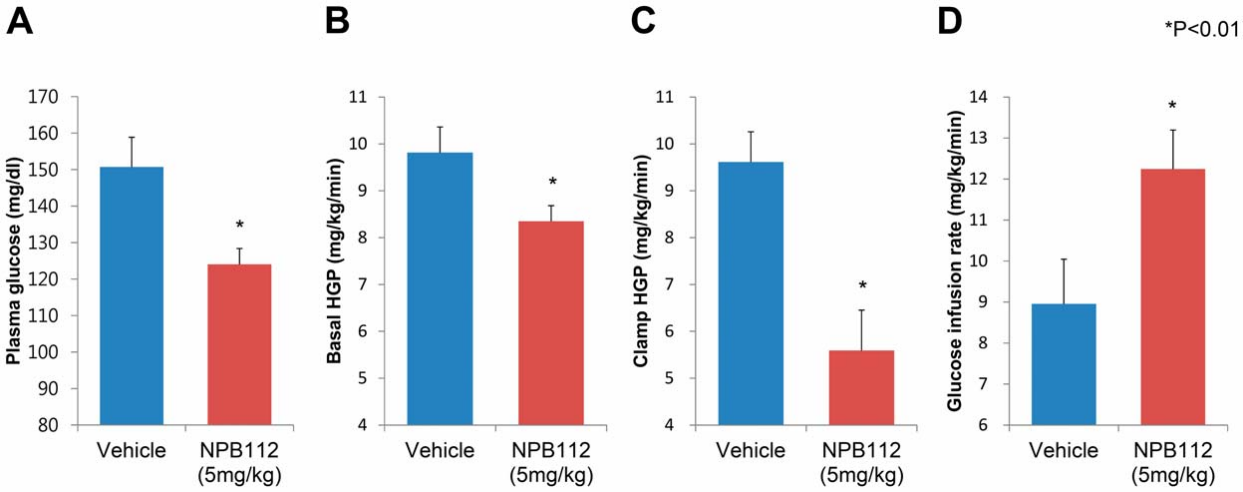
Effect of NPB112 on hepatic and peripheral insulin sensitivity in DIO mice. A. Fasting glucose levels in the hyperinsulinemic-euglycemic clamp studies before insulin infusion for mice treated with vehicle or NPB112 at 5.0 mg/kg. *n = 5* per group. B. Hepatic insulin action as the percent suppression of basal hepatic glucose production before insulin infusion for mice treated with vehicle or NPB112 at 5.0 mg/kg. *n = 5* per group. C. Hepatic insulin action as the percent suppression of clamp hepatic glucose production after insulin infusion at the rate of 15∼18 pmol/kg/min for mice treated with vehicle or NPB112 at 5.0 mg/kg. The clamp conditions were same as in A. *n = 5* per group. D. Steady-state whole-body glucose infusion rate (GIR) during the last 30 min of the hyperinsulinemic-euglycemic clamp studies in mice treated with vehicle or NPB112 at 5.0 mg/kg. *n = 5* per group. *, p<0.01 compared to control mice with vehicle.

As shown in Figure 4, control DIO mice treated with vehicle showed marked insulin resistance with the mean whole-body glucose infusion rate (GIR) at only 9.0 mg/kg/min. However, DIO mice with injection of NPB112 at 5.0 mg/kg were more insulin sensitive than control mice (the baseline whole-body GIR in the DIO mice with injection of NPB112 at 5.0 mg/kg was significantly increased), achieving a mean whole-body GIR of 12.0 mg/kg/min. This indicates that administration of NPB112 at a dose of 5.0 mg/kg provided ∼25% protection against HFD-induced insulin resistance.

With regard to hepatic insulin sensitivity, DIO mice treated with NPB112 showed a significant improvement in ability of insulin to suppress hepatic glucose production, demonstrating 33% suppression (from 8.3 mg/kg/min to 5.6 mg/kg/min) compared to the 2% suppression (from 9.8 mg/kg/min to 9.6 mg/kg/min) in control mice. In addition, treatment with NPB112 ameliorated hepatic insulin resistance induced by HFD in DIO mice, as reflected by a 15% reduction in basal hepatic glucose output in DIO mice treated with NPB112 at a dose of 5.0 mg/kg compared with control mice with vehicle. However, there was no significant difference in insulin-stimulated rate of whole-body glucose turnover, whole-body glycolysis, or whole-body glycogen synthesis between the two groups.

### Investigation of Adverse Effects of NPB112 on Hypoglycemia and other Laboratory Abnormalities

**Figure 5:**
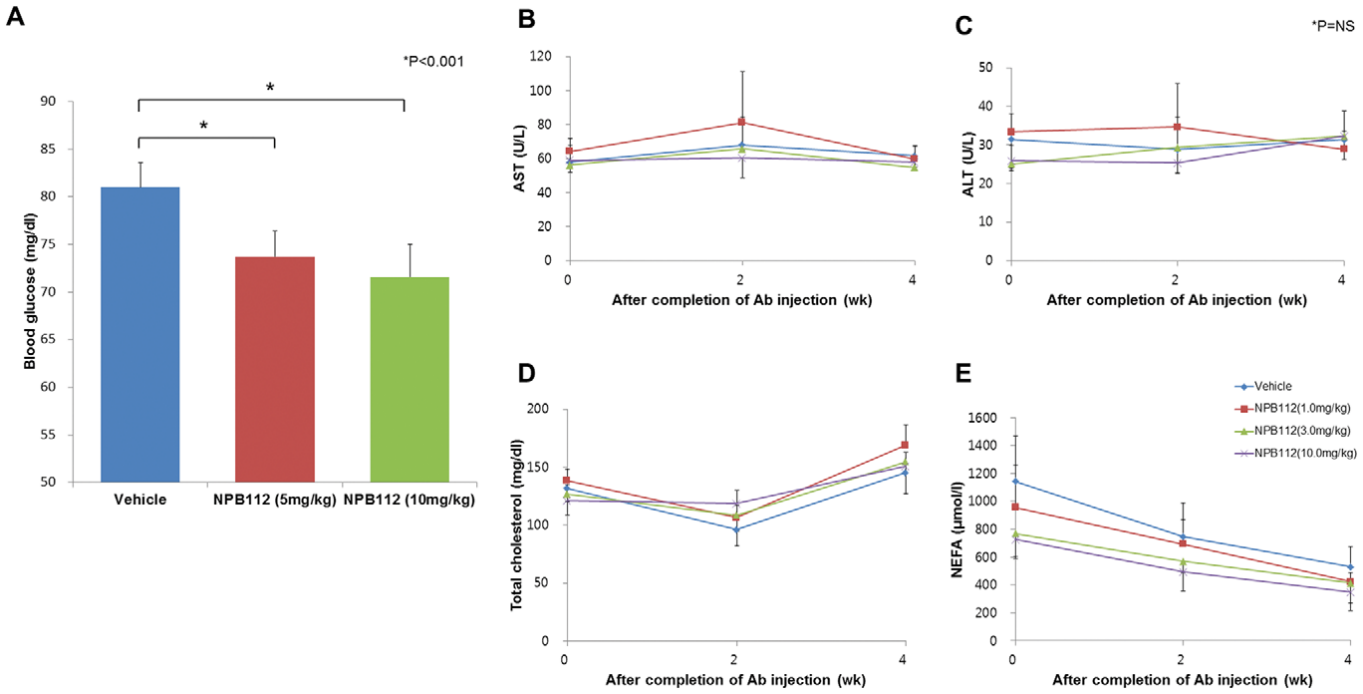
Effect of NPB112 on fasting plasma glucose (A) in normal C57B6 mice treated with NPB112 (n = 7 per group) and other laboratory profiles (B–E) in DIO mice before and every week after injection of vehicle or NPB112 for 6 weeks and 4 weeks of recovery (n = 24 per group). Values are expressed as mean ± SEM *, p<0.001 compared to control mice with vehicle.

To evaluate whether NPB112 induces hypoglycemia in normal conditions, we administrated NPB112 at a dose of 5.0 mg/kg or 10.0 mg/kg in normal ICR mice and measured plasma glucose levels after a 16-h fast, 48 h after injection of NPB112. ICR mice treated with NPB112 at 5.0 mg/kg and 10.0 mg/kg had significant 9% and 11% reductions in fasting plasma glucose level, respectively (Figure 5A), while no hypoglycemia (<60 mg/dL in FPG level) was observed in any of the animals.

To evaluate the adverse effect of NPB112 on DIO mice, liver enzymes such as AST, ALT and lipid profiles were measured 3 times in DIO mice treated with different doses of NPB112 and vehicle at the baseline, 2 weeks and 4 weeks after completion of NPB112 injection (during the recovery period). Compared to control mice treated with vehicle, there was no significant difference in levels of AST, ALT, total cholesterol, triglycerides or NEFA in DIO mice treated with different doses of NPB112 (Figures 5B–E).

## Discussion

Glucagon is an essential regulator of glucose homeostasis in both animals and humans. Contrary to the normal physiologic response (condition), plasma glucagon level is inappropriately elevated at a comparable level of blood glucose in subjects with T2D [15], while *α*-cell responsiveness to glucagon is nearly blunted or absent. Hyperglucagonemia and abnormal insulin-to-glucagon ratios observed in subjects with T2D contribute to pathologic hyperglycemia in both postabsorptive and fasting states by increasing hepatic glucose production [6], [16]. To date, although a number of drugs have been developed to control various targets related to the pathway of insulin secretion and insulin resistance, there is no commercially available medication that effectively modulates the glucagon-signaling pathway in humans.

In the present study, we developed a potent fully-humanized monoclonal antibody (NPB112) against GCGR, the 7-transmembrane G-protein-coupled receptor (class II or family B), using our proprietary techniques. Based on the physiologic action of glucagon, we hypothesized that the antibody against GCGR might effectively regulate glucagon-related glucose metabolism by suppressing the hepatic glucose output, resulting in decreasing glucose level during the fasting state and between meals. To demonstrate this hypothesis, we adopted the DIO mouse model with human GCGR in order to avoid interference from other hormones, such as insulin which is deficient in streptozotocin-induced type 1 diabetic models or leptin of which metabolism is impaired in *ob*/*ob* or *db*/*db* mice models. We therefore investigated whether (1) NPB112 binds and functionally antagonizes hGCGR *in vitro* and *in* *vivo*; (2) chronic treatment with NPB112 effectively lowers *in* *vivo* blood glucose level in diabetic animal models; (3) NPB112 effectively reduces the hepatic glucose output in those animals; and (4) prolonged treatment with NPB112 leads to elevation in glucagon or any unexpected adverse reactions including hypoglycemia or other laboratory abnormalities in hGCGR mice.

With respect to GCGR antagonist, several previous reports have demonstrated that GCGR is a plausible target for the treatment of T2D by using various substances including small-molecules (compound A, bicyclic peptide, NNC 25-0926) [7], [8], [17], [18], antisense oligonucleotides [9], [10] and monoclonal antibodies [11]–[13], [19]. The glucose-lowering effect of non-peptide receptor competitive antagonists, which are orally available, has been confirmed in various animal models including rodents [8], [18], [20], [21] and dogs [7]. Furthermore, a non-peptide compound with antagonistic activity against glucagon was developed and shown to effectively suppress glucagon-induced glucose production and subsequent hyperglycemia in normal human subjects [17]. However, traditional drug development with small molecular antagonists for GCGR has faced technical challenges associated with efficiency in blocking the ligand-receptor interaction packet characteristics of the family B GPCRs [22]. Regarding antisense oligonucleotides, chronic treatment with GCGR antisense oligonucleotides decreased blood glucose level as well as mRNA expression of GCGR and significantly improved glucose tolerance in diabetic rodent models such as *db*/*db*, *ob*/*ob* mice and ZDF rats [9], [10]. The antibody adopted in this study is a fully humanized monoclonal antibody antagonizing GCGR that offers a minimal risk of immunogenicity because it only contains antibody sequences of human origin, although this does raise possible concerns related to chimeric and humanized antibodies.

With respect to the efficacy and safety of NPB112, we demonstrated the following findings for this fully human monoclonal antibody (human IgG2): (1) NPB112 showed good affinity as assessed by cell ELISA and high functional activity confirmed by cAMP assay using a cell line (293-HEK-hGR-GFPcells) expressing mouse, cynomolgus monkey, and human glucagon receptors *in vitro*. Pharmacokinetic studies of NPB112 showed long period of half-life with low systemic clearance, suggesting an acceptable pharmacokinetic profile in the rat. (2) Six-week administration of NPB112 demonstrated a significant reduction in blood glucose. Despite being inferior to the anti-GCGR antibody reported by Yan H et al. [22], which maintained glucose-lowering effects for 8 days, a single intraperitoneal injection of NPB112 at 5 mg/kg effectively decreased the blood glucose level in DIO mice for 5 days. After repeated administration of NPB112 at dose of 3 or 10 mg/kg, the blood glucose levels were gradually elevated at 3 weeks of treatment, which might be attributed to the induction of anti-drug antibodies [23]. Because we injected fully-humanized monoclonal antibodies into DIO mice, the immune system of these animals would recognize NPB112 as a xenoantigen which leads to the generation of antibodies against NPB112 in the bloodstream after certain periods from initial injection. These findings are rooms for further experiments to analyze the characteristics of anti-drug antibodies. In addition, it is noteworthy that chronic treatment with NPB112 induced rather mild (∼1.5 fold increase) and reversible hyperglucagonemia in DIO mice. According to previous studies, hyperglucagonemia is the inevitable consequence of treatment with glucagon receptor antagonists [8]–[13], [19], [21]. In agreement with previous studies, 6-week administration of NPB112 in this study showed a significant reduction in blood glucose and concomitantly raised circulating glucagon level by 29% in DIO mice with hGCGR. With respect to changes of α cells which result in hyperglucagonemia, some studies reports that anti-glucagon receptor antagonists are known to induce mild to moderate hypertrophy and hyperplasia of α cells in islets [10], [13], [21], whereas the severity of α cells hyperplasia was much higher in GCGR KO mice [24], [25]. In contrast to these findings, the number and size of α cells were not significantly affected by the treatment with glucagon receptor antagonists using antisense oligonucleotides or non-peptide small molecules in several experiments [8], [9], although hyperglucagonemia was still observed. It is likely that blockade of glucagon signaling might interfere with the negative feedback-regulating pathway on glucagon secretion in α cells; however, no explanation has been offered for this mild hyperglucagonemia induced by NPB112. This might be due to the diversity in affinity to GCGR or an unidentified functional property of our antibody. (3) Regarding the effect of glucagon antagonist on weight, 6-week treatment with NPB112 caused mild body weight loss in DIO mice. It was reported that absence of glucagon signaling was associated with decreased body weight and food intake in GCGR knockout (*Gcgr−/−*) mice when HFD was challenged [24], implying a protective role of glucagon antagonists as anti-obesity agents. In light of the anti-obesity effect of GLP-1 [26], these findings might be attributed to an increased level of GLP-1 in blood with reduced gastric emptying in GCGR knockout mice. It has also been shown that treatment with glucagon receptor antagonist results in weight loss of DIO mice with no significant difference compared with control mice [21]. However, there are a few conflicting reports that GCGR antagonist did not cause significant weight loss in diabetic rodent models [8], [13], [19]. Therefore, further experiments regarding the extent of physical activity and food intake in diabetic animal models treated with glucagon antagonists should be carried out. (4) No hypoglycemia was observed in animals during short- and long-term treatment with NPB112, and even control mice fasted for 16 hours after injection of NPB112 had no evidence of hypoglycemia. In terms of adverse effects of NPB112, we did not observe any biochemical abnormalities such as elevation of AST or ALT level, or dyslipidemia after chronic treatment with NPB112 in DIO mice. To date, there are few published data on the adverse reactions related to glucagon antagonists. Treatment with antisense oligonucleotide significantly decreased plasma triglyceride levels as well as liver triglyceride contents in db/db mice and ZDF rats [10]. Liang et al. reported that a 3-week treatment with antisense oligonucleotide induced a significant decrease in serum levels of free fatty acids and triglyceride, while aspartate aminotransferase level was elevated in db/db mice, which might be linked to fatty change in the liver after this treatment [9]. Similar reduction in plasma triglycerides and NEFA was observed in our DIO animal models. Recent findings that glucagon receptor antagonist stimulated the fatty acid oxidation pathway [19] and enhanced insulin sensitivity of diabetic rodent models [19], [21] might suggest amelioration of increased levels of triglyceride and fatty acids in blood.

With respect to suppression of the hepatic glucose output by NPB112, DIO mice injected with NPB112 at 5 mg/kg showed a significant 33% improvement in terms of the ability of insulin to suppress HGP compared to the level at baseline. To demonstrate proof-of-concept that glucagon increased glucose level through HGP, we performed a euglycemic clamp study to clearly show that a single injection of our monoclonal antibody against GCGR significantly increased glucose infusion rate (GIR) with suppression of the rate of basal and clamp HGP, implying amelioration of peripheral insulin sensitivity in DIO mice. This finding suggests that the insulin sensitizing action of NPB112 is mainly mediated by its inhibition of production of glucose from liver by two major pathways – glycogenolysis and gluconeogenesis. Similar results were observed showing that an antibody against GCGR significantly reduced the rate of HGP by 45% in a hyperglycemic clamp study; however, basal HGP was not affected by the GCGR antibody [19]. This is in contrast to our findings that NPB112 significantly suppressed the basal HGP by 15% as well as the clamped HGP. Previous studies showed that the glucagon receptor antagonist, NNC 25-0926, effectively blocked HGP by inhibition of glycogenolysis in dogs [7]. Consistent with our data, Winzell et al. demonstrated that NNC 25-0926 significantly increased GIR in the euglycemic clamp study, indicating improvement of insulin sensitivity in DIO mice [21]. In this study, the inhibitory effect of antagonist on HGP was not further investigated, whereas insulin secretory function in islets was significantly improved after the treatment.

The present study has some limitations which should be overcome by further investigations. First, the pharmacokinetic and pharmacodynamics property of NPB112 were not fully examined. Second, measurement of blood level of GLP-1 and immunohistochemistry of pancreatic tissues were not performed to investigate whether NPB112 may stimulate GLP-1 secretion or cause histologic alterations such as *α*-cell hyperplasia in the pancreas and fatty changes in the liver. Third, physical activity and food intake in hGCGR mice were not analyzed. In addition, it would be more valuable to add a non-functioning monoclonal antibody as a negative control instead of using vehicles in the experiment.

In conclusion, human monoclonal antibody against GCGR significantly improved glucose homeostasis in diabetic mice by suppressing hepatic glucose output with no hypoglycemia or adverse events. These findings suggest that this anti-GCGR antibody could be a promising alternative treatment option for T2D in humans. Further studies including pharmacokinetics, pharmacodynamics and experiments with other T2D animal models are necessary to elucidate the underlying mechanism and to investigate additional therapeutic effects of NPB112 in T2D.
